# Association of high myopia with peripapillary retinal nerve fiber layer in patients with hypertension

**DOI:** 10.1371/journal.pone.0256131

**Published:** 2021-08-13

**Authors:** Min-Woo Lee, Hyung-Bin Lim, Hyung-Moon Koo, Young-Hoon Lee, Jung-Yeul Kim

**Affiliations:** 1 Department of Ophthalmology, Konyang University College of Medicine, Daejeon, Republic of Korea; 2 Department of Ophthalmology, Chungnam National University College of Medicine, Daejeon, Republic of Korea; Icahn School of Medicine at Mount Sinai, UNITED STATES

## Abstract

**Purpose:**

To identify the impacts of hypertension (HTN), high myopia, and the combination thereof on peripapillary retinal nerve fiber layer (pRNFL) thickness.

**Methods:**

All subjects were divided into four groups: control (group 1); patients with HTN without high myopia (group 2); patients with high myopia without HTN (group 3); and patients with both HTN and high myopia (group 4). The pRNFL thicknesses were compared using a one-way analysis of variance. Univariate and multivariate linear regression analyses were used to identify factors affecting pRNFL thickness in subjects with and without HTN.

**Results:**

The mean pRNFL thicknesses were 93.9±8.8, 88.7±6.8, 86.4±8.1, and 82.5±9.6 μm in group 1, 2, 3, and 4, respectively, and differed significantly (P<0.001). On multivariate linear regression analyses, age (β = -0.181, P = 0.044), axial length (β = -1.491, P<0.001), and HTN (β = -4.876, P = 0.044) significantly affected pRNFL thickness. Additionally, age and axial length affected the pRNFL thickness in subjects with HTN (age, β = -0.254, P = 0.020; axial length, β = -1.608, P<0.001) much more than in subjects without HTN (age, β = -0.028, P = 0.712; axial length, β = -1.324, P<0.001).

**Conclusions:**

High myopia and HTN affected pRNFL reduction and a combination of the 2 diseases exacerbated pRNFL damage. This could be a confounding factor in interpreting pRNFL thickness in patients with ophthalmic diseases affecting the pRNFL thickness when combined with the 2 diseases.

## Introduction

Hypertension (HTN), which causes cardiovascular, cerebrovascular, and renal disease, is known as a risk factor for various ophthalmic diseases such as retinal vascular occlusion, retinal macroaneurysms, and nonarteritic anterior ischemic optic neuropathy. HTN is also one of the risk factors for glaucoma. Many previous studies reported associations between HTN and thinning of the peripapillary retinal nerve fiber layer (pRNFL), which might be related to HTN as a risk factor for glaucoma [1,2]. Additionally, we recently reported that pRNFL thickness declined more markedly over time in patients with well-controlled HTN than in normal individuals [3]. In the study, axial length was a significant factor associated with pRNFL reduction in the HTN group. However, we could not study the detailed association between pRNFL reduction and axial length in patients with HTN because we did not enroll eyes with axial length≥26.0 mm.

High myopia, defined as an axial length≥26.0 mm, can cause chorioretinal atrophy, choroidal neovascularization, or macular retinoschisis because the structure of highly myopic eyes becomes thinner via fewer cross-linkage [4]. Additionally, previous studies reported that myopic eyes showed a consistent increase in axial length [5,6]. Such structural changes could affect retinal layer thickness in high myopia. Many previous studies reported that myopia has thinner inner retina than normal individuals [7–9]. Our previous studies also identified greater reductions in pRNFL and ganglion cell-inner plexiform layer (GC-IPL) over time in patients with high myopia compared to normal individuals [10,11].

HTN and high myopia, which are relatively common and increasing in prevalence, are both associated with pRNFL reductions as mentioned above [12,13]. However, how these 2 diseases interact in terms of pRNFL thickness has not been reported as far as we know. In this study, we evaluated the pRNFL thickness in eyes with and without high myopia of patients with and without HTN.

## Methods

### Patients

This retrospective, cross-sectional study adhered to the tenets of the Declaration of Helsinki and was approved by the Institutional Review Board of Chungnam National University Hospital, Daejeon, Republic of Korea. We reviewed the charts of patients who visited our retina clinic from March 2011 to July 2019; we investigated the detailed history, best-corrected visual acuity (BCVA), intraocular pressure (IOP), spherical equivalent (SE), and axial length (using an IOLMaster; Carl Zeiss, Jena, Germany, version 5.02) of each patient. The sample size was calculated using ‘G power’ version 3.1 sample size package with a setting of a = 0.05 and statistical power = 0.8. The requirement for obtaining informed consent was waived due to the retrospective nature of the study. The subjects were divided into four groups: control (group 1), patients with HTN without high myopia (group 2), patients with high myopia without HTN (group 3), and patients with HTN and high myopia (group 4). We defined high myopia as an axial length≥26.0 mm and enrolled patients with well-controlled HTN≥5 years in duration. We excluded subjects with systolic blood pressure≥140 or diastolic blood pressure≥90 mmHg. The exclusion criteria were a medical history of diabetes, any ophthalmic disease that can affect the thickness of the retinal layer, such as glaucoma, retinal diseases, neuro-ophthalmic diseases, any prior intraocular surgery except cataract extraction, a BCVA<30/40, and an IOP>21 mmHg. We also excluded patients with high myopia with structural changes such as large chorioretinal atrophy or posterior staphyloma, which could cause segmentation error when measuring pRNFL thickness.

### OCT measurements

OCT measurements were performed by a skilled examiner using a Cirrus HD OCT 5000 (Carl Zeiss Meditec, Dublin, CA; version 10.0). pRNFL thickness was measured using the 200x200 optic disc cube scanning protocol. The optic nerve head was placed at the center of the scanned image, and a 200x200-pixel resolution axial scan was performed over an area of 6x6 mm that included the optic nerve head and its surroundings. Images with a signal strength<7, any motion artifact, an involuntary saccade, obvious decentration misalignment, or a segmentation error were excluded.

### Statistical analysis

After applying the Kolmogorov-Smirnov test of normality, demographic characteristics, ocular parameters, and pRNFL thicknesses were compared using one-way analysis of variance with the post-hoc Bonferroni correction, and the chi-squared test. Analysis of covariance was used to compare the pRNFL thicknesses among groups after adjusting for age, sex, IOP, BCVA, and central macular thickness (CMT). Patients with HTN were classified into four subgroups, reflecting the most commonly used regimens of antihypertensive treatment: (A) angiotensin-converting enzyme inhibitors and/or angiotensin-receptor blockers; (B) beta-blockers and/or calcium-channel blockers; (C) diuretics alone or combined with other medications; and (D) other combinations [14,15]. Univariate and multivariate linear regression analyses were performed to identify factors affecting the pRNFL thickness. Statistical analyses were performed using SPSS version 18.0 software (IBM Copr., Armonk, NY).

## Results

### Demographics

A total of 164 eyes were enrolled: 50 eyes in group 1, 43 eyes in group 2, 41 eyes in group 3, and 30 eyes in group 4. The mean age was 60.7±6.1, 63.3±6.1, 60.2±5.8, and 63.1±12.1 years in the group 1, 2, 3, and 4, respectively, which was not significantly different (P = 0.144) (Table 1).

**Table 1:** Demographics and clinical characteristics.

Characteristics	Group 1 (n = 50)	Group 2 (n = 43)	Group 3 (n = 41)	Group 4 (n = 30)	P value
Age, years	60.7±6.1	63.3±6.1	60.2±5.8	63.1±12.1	0.144
Sex, male (%)	22 (44%)	23 (53.5%)	15 (36.6%)	12 (40.0%)	0.672
Laterality, right (%)	20 (40%)	22 (51.2%)	22 (53.7%)	14 (46.7%)	0.812
IOP, mmHg	15.4±2.4	15.5±2.0	16.3±2.3	16.2±3.0	0.170
BCVA, logMAR	0.004±0.019	0.003±0.037	0.012±0.063	0.020±0.045	0.311
SE, diopters	-0.29±1.31	0.09±1.15	-5.81±8.38	-6.30±5.97	**<0.001**
Axial length, mm	23.84±1.00	23.74±0.89	28.23±2.03	27.86±1.87	**<0.001**
HTN duration, years	N/A	15.8± 7.5	N/A	13.1± 5.9	0.103
SBP, mmHg	117.3±9.9	122.1±7.1	116.8±10.5	123.4±8.0	0.130
DBP, mmHg	72.8±8.0	74.2±6.7	73.5±7.0	73.9±8.3	0.524
CMT, μm	250.1±24.5	253.1±21.8	261.6±20.2	254.2±23.6	0.110

IOP, intraocular pressure; BCVA, best-corrected visual acuity; SE, spherical equivalent; HTN, hypertension; SBP, systolic blood pressure; DBP, diastolic blood pressure; CMT, central macular thickness.

Group 1, control; group 2, patients with hypertension without high myopia; group 3, patients with high myopia without hypertension; group 4, patients with both hypertension and high myopia.

Data are the mean ± standard deviation.

Boldface numbers indicate statistically significant differences at P<0.05.

Sex, laterality, blood pressure, IOP, and BCVA were also not significantly different among the 4 groups. SE and axial length showed significant differences among groups. In post hoc analyses, SE and axial length did not show significant differences between group 1 and 2 (P = 0.983 and P = 0.978), and between group 3 and 4 (P = 0.988 and P = 0.736). In subgroups classified according to the HTN medication, the number of subgroup A, B, C, and D was 9 (20.9%), 9 (20.9%), 13 (30.2%), and 12 (27.9%) in group 2, and 6 (20.0%), 8 (26.7%), 7 (23.3%), and 9 (30.0%) in group 4, which was not significantly different between the two groups (P = 0.896).

### pRNFL thickness of each group

The mean pRNFL thicknesses were 93.9±8.8, 88.7±6.8, 86.4±8.1, and 82.5±9.6 μm in group 1, 2, 3, and 4, respectively, and differed significantly (P<0.001) (Table 2).

**Table 2:** Peripapillary retinal nerve fiber layer (pRNFL) thickness of each group.

	Group 1 (n = 50)	Group 2 (n = 43)	Group 3 (n = 41)	Group 4 (n = 30)	P value	Post-hoc analyses
pRNFL						
Mean	93.9±8.8	88.7±6.8	86.4±8.1	82.5±9.6	**<0.001**	Group 1>2>4
Sector						
Superior	115.4±14.7	111.8±14.7	99.8±15.0	93.5±19.4	**<0.001**	Group 1, 2>3, 4
Nasal	66.5±8.1	66.0±7.7	67.5±8.9	67.5±11.5	0.828	
Inferior	123.7±15.1	115.5±16.3	99.0±19.2	96.0±19.1	**<0.001**	Group 1, 2>3, 4
Temporal	69.5±8.6	67.6±10.0	77.1±12.2	74.7±12.7	**<0.001**	Group 3, 4>1, 2

Group 1, control; group 2, patients with hypertension without high myopia; group 3, patients with high myopia without hypertension; group 4, patients with both hypertension and high myopia.

Data are the mean±standard variation (μm).

P value for one-way analysis of variance followed by post-hoc Bonferroni correction.

Boldface numbers indicate statistically significant differences at p<0.05.

On post-hoc analysis, group 1 showed a significant difference from all the other groups (vs group 2, P = 0.014; vs group 3 and 4, both P<0.001). Group 2 did not differ significantly from group 3 (P = 0.602) but did from group 4 (P = 0.014). Group 3 did not show a significant difference from group 4 (P = 0.223). In the sectoral analyses, the superior, inferior, and temporal sectors differed significantly between the 4 groups (all P<0.001), but the nasal sector did not(P = 0.828). After adjusting for covariants, the estimated mean pRNFL thicknesses of the 4 groups were 93.5±1.2, 88.7±1.3, 86.4±1.3, and 83.1±1.6 μm, respectively (P<0.001), and the post hoc analysis yielded similar results (Table 3). In comparison between group 3 and group 4, we adjusted for covariants including axial length because of the difference in axial length between the 2 groups, and there were significant differences in mean pRNFL thickness (P = 0.033) and the thickness of the superior sector (P = 0.038).

**Table 3:** Estimated mean peripapillary retinal nerve fiber layer (pRNFL) thickness after adjusting for covariants.

	Group 1 (n = 50)	Group 2 (n = 43)	Group 3 (n = 41)	Group 4 (n = 30)	P value	Post-hoc analyses
pRNFL						
Mean	93.5±1.2	88.7±1.3	86.4±1.3	83.1±1.6	**<0.001**	Group 1>2> 4
Sector						
Superior	114.4±2.1	111.5±2.3	99.9±2.4	95.4±2.9	**<0.001**	Group 1, 2>3, 4
Nasal	66.5±1.3	65.7±1.4	67.9±1.4	67.2±1.7	0.703	
Inferior	123.2±2.4	115.6±2.7	99.1±2.7	96.7±3.3	**<0.001**	Group 1, 2>3, 4
Temporal	69.5±1.6	67.6±1.7	77.3±1.7	74.6±2.1	**<0.001**	Group 1, 2>3, 4

Group 1, control; group 2, patients with hypertension without high myopia; group 3, patients with high myopia without hypertension; group 4, patients with both hypertension and high myopia.

Data are the mean±standard error (μm).

P value for analysis of covariance with the post-hoc Bonferroni correction.

Boldface numbers indicate statistically significant differences at P<0.05.

### Linear regression analyses between various clinical factors and pRNFL thickness

In univariate analyses, age (β = -0.192, P = 0.048), IOP (β = -0.678, P = 0.026), BCVA (β = -35.220, P = 0.035), axial length (β = -1.445, P<0.001), HTN (β = -4.256, P = 0.003), and HTN duration (β = -0.175, P = 0.036) significantly affected pRNFL thickness (Table 4).

**Table 4:** Univariate and multivariate linear regression analyses between various clinical factors and peripapillary retinal nerve fiber layer thickness.

	Univariate regression		Multivariate regression	
	β	P value	β	P value
Age	-0.192 (-0.381, -0.002)	**0.048**	-0.181 (-0.356, -0.005)	**0.030**
Sex	-0.717 (-3.593, 2.158)	0.623		
IOP	-0.678 (-1.272, -0.084)	**0.026**	-0.260 (-0.805, 0.285)	0.333
SE	0.316 (0.071, 0.560)	**0.012**		
BCVA	-35.220 (-67.965, -2.482)	**0.035**	-5.302 (-36.043, 25.439)	0.734
Axial length	-1.445 (-1.951, -0.939)	**<0.001**	-1.491 (-2.019, -0.964)	**<0.001**
HTN	-4.256 (-7.050, -1.462)	**0.003**	-4.876 (-9.620, -0.131)	**0.001**
HTN duration	-0.175 (-0.339, -0.012)	**0.036**	0.026 (-0.253, 0.305)	0.853
SBP	-0.179 (-0.399, 0.040)	0.108		
DBP	-0.040 (-0.338, 0.257)	0.789		
CMT	0.030 (-0.033, 0.092)	0.349		

IOP, intraocular pressure; SE, spherical equivalent; BCVA, best-corrected visual acuity; HTN, hypertension; SBP, systolic blood pressure; DBP, diastolic blood pressure; CMT, central macular thickness.

Boldface numbers indicate statistically significant differences at P<0.05.

**Fig 1:**
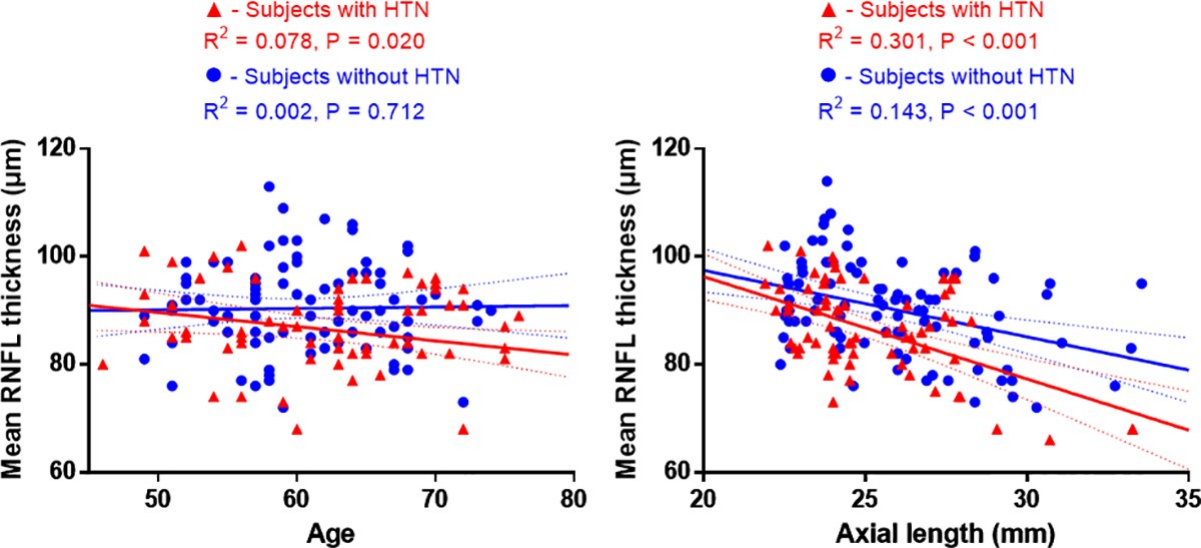
Scatterplots and linear regression analyses between the mean peripapillary retinal nerve fiber layer thickness (pRNFL) and age and axial length in subjects with hypertension (HTN) and subjects without HTN. The pRNFL thickness of subjects with HTN showed a much stronger correlation with age and axial length than that of subjects without HTN.

Among these factors, age vs. HTN, age vs. HTN duration, and HTN vs. HTN duration showed significant interaction, but their variance inflation factors were lower than 4, which means low collinearity. In multivariate analyses, age (β = -0.181, P = 0.030), axial length (β = -1.491, P<0.001), and HTN (β = -4.876, P = 0.001) showed significant results. Additionally, age and axial length affected pRNFL thickness in subjects with HTN (age, β = -0.254, P = 0.020; axial length, β = -1.608, P<0.001) to a much greater extent than in subjects without HTN (age, β = -0.028, P = 0.712; axial length, β = -1.324, P < 0.001) (Fig 1). In patients with HTN, antihypertensive medication according to the subgroup classification did not significantly affect the pRNFL thickness (β = -0.023, P = 0.981).

## Discussion

Previous studies reported inner retinal changes in patients with HTN without any ophthalmic disease [1–3,16,17]. Mauschitz et al. [1] reported reduced pRNFL thickness in hypertensive patients without the association of actual systolic blood pressure (β = -0.54 μm; 95% confidence interval, -1.01 to -0.07). Another study reported that the pRNFL of hypertensive patients was thinner than healthy controls, especially in the superior and inferior quadrants [17]. Such changes are also observed in eyes with high myopia. Lamparter et al. [18] reported the association of a thinner pRNFL with myopia and a longer axial length using spectralis-OCT. Leung et al. [19] also reported that the average RNFL thickness decreased with increasing axial length (r = -0.314, P = 0.001) and negative refractive power (r = 0.291, P = 0.002). These changes could be a confounding factor for diagnosis or follow-up of diseases such as glaucoma. Therefore, when interpreting pRNFL thicknesses in patients with HTN or high myopia, changes associated with these 2 diseases themselves should be considered. However, the study of pRNFL thickness in patients with both diseases has not been reported as far as we know. We identified that patients with both diseases tended to have thinner pRNFL than patients with only 1 disease.

Our previous study identified marked reductions of pRNFL thickness in the superior and inferior quadrants of HTN patients compared to normal controls [3]. Sahin et al. [17] also reported a significant difference in superior and inferior quadrants between HTN patients and healthy controls (superior quadrant, 106.9±17.5 vs 118.9±14.7 μm, P<0.001; inferior quadrant, 109.0±18.7 vs 118.6±13.8 μm, P = 0.003). They explained that secondary hypertensive vascular changes in retinal vessels could cause disturbance of blood flow autoregulation, resulting in the optic nerve prone to perfusion deficiency and retinal ischemia; such changes affected particularly the superior and inferior quadrants of the retinal nerve in HTN. Our study showed a similar tendency showing thinner pRNFL thickness in superior and inferior sectors of group 2 than group 1. We hypothesize that superior and inferior sectors, which have relatively thicker pRNFL than the temporal and nasal sectors, would have higher oxygen demands than the latter sectors, rendering them more sensitive to ischemic damage. Although group 1 and 2 did not show a significant difference on post-hoc analyses, subjects with HTN showed a significant negative relationship between pRNFL thickness and age (β = -0.254, P = 0.020). Thus, the differences in the pRNFL thicknesses of the superior and inferior sectors might get prominent over time between patients with HTN and normal individuals.

Group 3 showed thinner mean pRNFL than group 1. In terms of sectoral pRNFL thicknesses, group 3 exhibited significantly thinner superior and inferior sectors, and a significantly thicker temporal sector than group 1, as in previous studies [11,20,21]. The thicker pRNFL of the temporal area in Group 3 would be related to a high percentage of peak RNFL thicknesses with temporal deviation in high myopia [20]. This temporal deviation in the peak RNFL thickness may cause temporal RNFL thickening in high myopia. Group 3 had a somewhat thinner mean pRNFL than group 2, although it was not significant in post-hoc analyses (P = 0.602). Whereas, group 3 had significantly thinner superior and inferior sectors than group 2 (P = 0.004 and P < 0.001). pRNFL damage to the superior and inferior sectors due to the mechanical stretching in high myopia might affect pRNFL reduction more than the damage due to the retinal ischemia in HTN. This result could be caused by the difference in beginning time and duration of pRNFL damage between the 2 diseases. The mechanical stretching in high myopia would affect the pRNFL from a young age, whereas the retinal ischemia in HTN would begin to damage the pRNFL a few decades later than high myopia. However, further histopathological studies identifying the exact mechanism of pRNFL damage caused by 2 diseases are needed.

Group 4 differed significantly from group 1 and 2 in terms of both mean and sectoral pRNFL thicknesses, except in the nasal sector. Group 4 also tended to have thinner mean and sectoral pRNFL thicknesses than group 3, which showed a significant difference in mean and superior pRNFL thickness after adjusting the axial length. In subjects with HTN, pRNFL thicknesses were more affected by age than in subjects without HTN. The previous study also reported that age was a significant factor associated with the reduction in pRNFL thickness in HTN (estimate, -0.362 μm; P = 0.042) [3]. Therefore, subjects with older age might show a significant difference in pRNFL thickness between group 3 and 4. However, further longitudinal studies with a large number of cases are needed. Nevertheless, we could hypothesize that a combination of mechanical stretching and ischemic damages would affect the pRNFL reduction more than ischemic or mechanical damage alone; the 2 mechanisms may be synergistic. Additionally, both mechanisms could become more intense over time by the accumulation of atherosclerotic changes in the arteries and constant elongation of axial length. Thus, physicians should consider these findings when interpreting pRNFL changes in subjects with both diseases and, indeed, other ophthalmic diseases such as glaucoma.

To identify in more detail how axial length affected pRNFL thickness in HTN patients, we examined the relationship between pRNFL thickness and axial length in subjects with HTN and subjects without HTN. In both groups, the longer the axial length, the thinner the pRNFL. However, subjects with HTN had a much stronger negative relationship between axial length and pRNFL thickness than subjects without HTN. Our previous study reported that axial length significantly affected the reduction in pRNFL thickness in HTN patients [3]. In the study, we did not enroll the subjects with axial length≥26.0 mm because we sought to exclude conditions other than HTN that might affect pRNFL thickness. From the result of the present study, we found that even an axial length≥26.0 mm affected the reduction in pRNFL to a greater extent in patients with HTN than in subjects without HTN.

### Study limitations and strengths

Apart from the retrospective nature of the work, this study has several limitations. First, we could not totally rule out the possibility that there had been an occurrence and regression of hypertensive retinopathy in HTN patients in the past. Second, we could not identify the relationship between various factors such as nocturnal blood pressure dips and pRNFL thickness in HTN patients because of limited information by retrospective characteristics. Third, a relatively small number of group 4 may cause some bias. Fourth, our study could not definitely determine whether HTN or antihypertensive medication would have a greater impact on RNFL thinning. The strength of our study is that we identified relationships between pRNFL thickness and HTN, high myopia, and the combination of the 2 diseases, which has not been reported as far as we know.

## Conclusions

In conclusion, high myopia and HTN affected the thin pRNFL thickness and a combination of the 2 diseases intensified pRNFL damage. This is in line with that axial length affects the pRNFL thickness in patients with HTN much more than in normal individuals. The mechanisms of pRNFL thinning in 2 diseases, mechanical stretching and retinal ischemia, might operate synergistically, becoming more severe over time by the progression of atherosclerosis in retinal vessels and constant elongation of the axial length. Therefore, physicians should be aware of greater pRNFL damage by the coexistence of 2 diseases, which are very common and both increasing their prevalence, and careful observations for changes in pRNFL thickness are needed regularly. Additionally, physicians should also consider the RNFL change by these 2 diseases themselves when interpreting pRNFL changes in patients with diseases affecting pRNFL such as glaucoma.

## Supporting information

S1 Data (XLSX)
